# Perirenal Hematomas Induced by Extracorporeal Shock Wave Lithotripsy (ESWL). Therapeutic Management

**DOI:** 10.1100/tsw.2007.236

**Published:** 2007-09-17

**Authors:** Apostolos P. Labanaris, Reinhard Kühn, Günter E. Schott, Vahudin Zugor

**Affiliations:** ^1^ Department of Urology, Martha Maria Medical Center, Academic Hospital of Erlangen University, Nurnberg, Germany; ^2^ Department of Urology, University of Erlangen Medical Center, Erlangen, Germany

**Keywords:** ESWL, perirenal hematoma, conservative management

## Abstract

Extracorporeal shock wave lithotripsy (ESWL) is nowadays accepted as the treatment of choice for the majority of patients with renal or proximal ureteral calculi. Although, a relatively noninvasive modality with low morbidity, minor or major complications can be noted. A relative severe complication for the patient and confusing for the treating physician is the perirenal hematoma. With review the literature and an example of perirenal hematoma induced by ESWL in a patient treated in our department, we describe its therapeutic management.

## INTRODUCTION

Extracorporeal shock wave lithotripsy (ESWL) is an effective, relatively noninvasive modality with low morbidity for the treatment of upper urinary tract calculi. First described clinically by Chaussy and associates in 1980[1], it is now accepted as the treatment of choice for the majority of patients with renal or proximal ureteral calculi. Numerous studies have been focused on the effectiveness, side effects, and complications of ESWL treatment. It is thought that the mechanism that plays a role in the development of the complications is the compressive and tensile forces of shockwaves directed at soft tissue, and the effect of cavitation on the individual cell integrity[2]. Although the majority of complications relate to the passage of stone fragments, including severe pain and ureteral obstruction, renal or perirenal hematomas are a well-known complication of ESWL.

We review the literature and present an example of perirenal hematoma induced by ESWL and its therapeutic management.

## CASE REPORT

In January 2006, a 46-year-old man presented to our department with a history of a left kidney stone and was scheduled for ESWL. His past medical history revealed no contraindications to ESWL, which include bleeding tendencies, uncontrolled hypertension, active urinary tract infection, kidney disease, morbid obesity, or anatomic abnormalities of the urinary tract. The procedure was performed with a permanently installed third-generation lithotripter. After intravenous sedation of the patient, ESWL treatment began with a final shockwave number of 3000 and a voltage range of 15 kV. During the procedure and the first 24 h afterwards, no pain or gross hematuria were reported by the patient. The following day, the patient reported mild pain in the left flank. The patient denied nausea, vomiting, chest pain, or shortness of breath. Physical examination revealed a middle-aged man in moderate distress due to pain. Vital signs were: temperature, 36.6°C; blood pressure, 121/71 mm Hg; heart rate, 68 beats/min; and respiratory rate, 18 breaths/min. The heart was regular without murmurs and lungs were clear to auscultation. The abdomen was soft and nontender to palpation. Back examination revealed left flank percussion tenderness at the costovertebral angle. No bruising was present. Laboratory studies revealed hemoglobin of 12.1 g/dl and a white blood cell count of 14 k/Ul. Urinalysis was significant for 100–150 red blood cells. Sonography revealed a large hematoma of the left kidney with probable rupture into the retroperitoneum. Due to the sonographic results, a computed tomography (CT) scan of the abdomen was ordered (Fig. 1). The CT scan verified the sonographic results. Creatinine levels were pre- and postoperative 0.8 and 0.76 mg/dl, respectively. The size of the hematoma was approximately 12 × 16 × 8 cm. Over the following 36 h, the patient was noted to have a drop in his hemoglobin to 7.7 g/dl. Blood transfusion was required. On the third hospital day, the patient became hemodynamically stable with a rise in his hemoglobin to 11.3 g/dl. A repeat CT scan of the abdomen did not reveal change in the size of the hematoma. By the fifth hospital day, the hemoglobin became stable and the patient's pain had significantly improved. He was discharged from the hospital without any kind of surgical intervention. Up to the present day, the patient has not presented hypertension secondary to renal compression or abnormal creatinine levels.

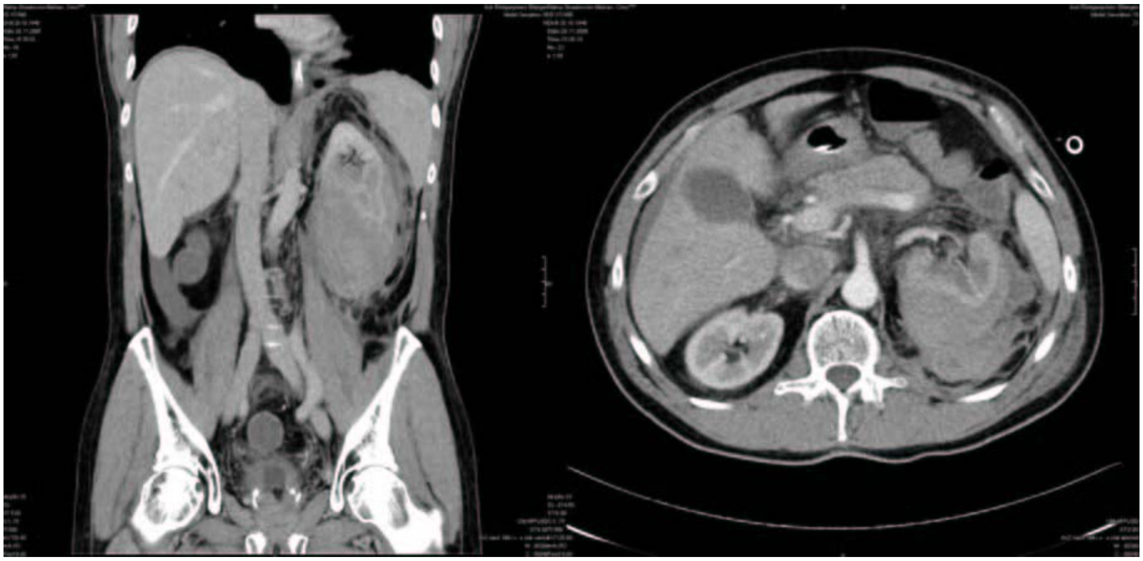

**FIGURE 1**. CT of a left perirenal hematoma.

## DISCUSSION

ESWL is an effective, relatively noninvasive modality accepted as the treatment of choice for the majority of patients with renal or proximal ureteral calculi. Nevertheless, minor or major complications can be noted. In order to avoid or to lower the incidence of these complications, proper selection of the candidates should be achieved with strict respect to the contraindications to ESWL. These include bleeding tendencies, pregnancy, uncontrolled hypertension, active urinary tract infection, morbid obesity, and anatomic abnormalities of the urinary tract. Major complications following ESWL are rare. The most frequent effect is microhematuria. ESWL treatment induces microtrauma and microscopic hemorrhage in normal kidneys[3,4,5]. Perinephric hematoma formation and infection, including acute pyelonephritis, are other known complications[2]. Indeed, the risk of peri- or intrarenal hematomas is estimated to be between 0.1 and 0.6% using ultrasonography[6,7,8], and between 20 and 25% using magnetic resonance imaging or CT[9,10]. The incidence, though, of clinically significant hemorrhage is less than 1%[11].

No clear correlation has been demonstrated between the number of shockwaves or the intensity supplied and the incidence of hematomas[9]. Predisposing factors include untreated arterial hypertension, coagulation deficits, and the use of antiplatelet drugs[11,12,13].

In the majority of cases of post-ESWL subcapsular hematomas, renal function is preserved and spontaneous radiographic resolution occurs within 2 years[14]. Coagulation disorders seem to correlate with the size of the hematoma rather than with the risk of its development[15].

The key to successful management of patients with renal trauma is an accurate assessment of the extent of renal injury. Proponents of a nonoperative approach believe that the majority of renal parenchymal injuries will heal without surgical exploration[16,17]. Skolarikos et al.[18] performed a literature search with the Medline database on ESWL between 1980 and 2004, reviewing 3,937 abstracts and reading 220 papers in full. They noted that treatment of the hematomas is conservative in most cases. The most likely outcome is spontaneous radiographic resolution of the hematoma within 2 years without clinically evident adverse effects on blood pressure or renal function.

Emergency renal exploration will still be required in the presence of hemodynamic instability despite appropriate resuscitation[19]. The most important late complication of a nonoperative approach is the Page kidney. The term “Page kidney” refers to the occurrence of hypertension secondary to renal compression usually associated with a perinephric or subcapsular hematoma. This phenomenon was first described by Page in 1939[20]. The first clinical case was described in 1955, in a patient who developed hypertension and was subsequently found to have a subcapsular hematoma[21].

The underlying pathophysiology is thought to result from renal ischemia and hypoperfusion, which consequently activates the renin-angiotensin-aldosterone system[22]. There are many possible causes of Page kidney, such as hematomas associated with trauma, iatrogenic, anticoagulants, and even associated with nonhematomas, such as large simple cysts. The time from injury to the onset of hypertension ranges from days to decades, with a mean of 36 months[23].

There is some evidence to suggest that renal trauma-induced hypertension is probably overestimated, and it is difficult to know if pre-existing essential hypertension might be a contributory factor[24].

At present, there are no definitive guidelines for the management of Page kidney. Previously, the management focused on surgical intervention. However, nowadays, conservative management is the usual recommended first-line treatment in such cases. With improvements in the range of oral antihypertensives, conservative treatment is more efficient and can provide a good quality of life for the patient[25]. In the event of persistent hypertension that is not responsive to treatment, surgical decompression might be required. There are two main aims of treatment: to decompress the subcapsular hematoma and to remove the fibrocollagenous shell.

## CONCLUSION

Renal and perirenal hematomas induced by ESWL should be managed with a nonoperative approach, since the majority of renal parenchymal injuries will heal without surgical exploration. Emergency renal exploration is required only in the presence of hemodynamic instability despite appropriate resuscitation.
